# A Generalized Formal Semantic Framework for Smart Contracts

**DOI:** 10.1007/978-3-030-45234-6_4

**Published:** 2020-03-13

**Authors:** Jiao Jiao, Shang-Wei Lin, Jun Sun

**Affiliations:** 8 University of Paderborn, Paderborn, Germany; 9 ICREA, Open University of Catalonia, Barcelona, Spain; 10 Nanyang Technological University, Singapore, Singapore; 11 Singapore Management University, Singapore, Singapore

**Keywords:** Blockchain, Smart contracts, Generalized semantics

## Abstract

Smart contracts can be regarded as one of the most popular blockchain-based applications. The decentralized nature of the blockchain introduces vulnerabilities absent in other programs. Furthermore, it is very difficult, if not impossible, to patch a smart contract after it has been deployed. Therefore, smart contracts must be formally verified before they are deployed on the blockchain to avoid attacks exploiting these vulnerabilities. There is a recent surge of interest in analyzing and verifying smart contracts. While most of the existing works either focus on EVM bytecode or translate Solidity contracts into programs in intermediate languages for analysis and verification, we believe that a direct executable formal semantics of the high-level programming language of smart contracts is necessary to guarantee the validity of the verification. In this work, we propose a generalized formal semantic framework based on a general semantic model of smart contracts. Furthermore, this framework can directly handle smart contracts written in different high-level programming languages through semantic extensions and facilitates the formal verification of security properties with the generated semantics.

## 1 Introduction

Blockchain [17] technologies have been studied extensively recently. Smart contracts [16] can be regarded as one of the most popular blockchain-based applications. Due to the very nature of the blockchain, credible and traceable transactions are allowed through smart contracts without relying on an external trusted authority to achieve consensus. However, the unique features of the blockchain introduce vulnerabilities [10] absent in other programs.

Smart contracts must be verified for multiple reasons. Firstly, due to the decentralized nature of the blockchain, smart contracts are different from programs written in other programming languages (e.g., C/Java). For instance, the storage of each contract instance is at a permanent address on the blockchain. In this way, each instance is a particular execution context and context switches are possible through external calls. Particularly, in Solidity, `delegatecall` executes programs in the context of the caller rather than the recipient, making it possible to modify the state of the caller. Programmers must be aware of the execution context of each statement to guarantee the programming correctness. Therefore, programming smart contracts is error-prone without a proper understanding of the underlying semantic model. Secondly, a smart contract can be deployed on the blockchain by any user in the network. Vulnerabilities in deployed contracts can be exploited to launch attacks that lead to huge financial loss. Verifying smart contracts against such vulnerabilities is crucial for protecting digital assets. One famous attack on smart contracts is the DAO attack [41] in which the attacker exploited the reentrancy vulnerability and managed to take 60 million dollars under his control. Thirdly, it is very difficult, if not impossible, to patch a smart contract once it is deployed due to the very nature of the blockchain.

**Related Works.** There is a surge of interest in analyzing and verifying smart contracts [32,12,24,28,26,9,25,31,21,44,20,22,38,36,4,34,43,19,30,35,29,23,46,14]. Some of the existing works focus on EVM [2,47] (Ethereum Virtual Machine). For instance, a symbolic execution engine called Oyente is proposed in [32] to analyze Solidity smart contracts by translating them into EVM bytecode. In addition, a complete formal executable semantics of EVM [24] is developed in the K-framework to facilitate the formal verification of smart contracts at bytecode level. A set of test oracles is defined in [26,45] to detect security vulnerabilities on EVM bytecode. In [21], a semantic framework is proposed to analyze smart contracts at EVM level. Securify [44] translates EVM bytecode into a stackless representation in static-single assignment form for analyzing smart contracts. In other works, Solidity smart contracts are translated into programs in intermediate languages for analysis and verification. Specifically speaking, Solidity programs are formalized with an abstract language and then translated into LLVM bitcode in Zeus [28]. Similarly, Boogie is used to verify smart contracts as an intermediate language in the proposed verifiers in [31,23]. In addition, the formalization in F* [12] is an intermediate-level language for the equivalence checking of Solidity programs and EVM bytecode. In [22], a simple imperative object-based programming language, called SMAC, is used to facilitate the online detection of Effectively Callback Free (ECF) objects in smart contracts. To conclude, most of the existing approaches either focus on EVM bytecode, or translate Solidity smart contracts into programs in intermediate languages that are suitable for verifying smart contracts or detecting potential issues in associated verifiers or checkers. Furthermore, none of the existing works can directly handle smart contracts written in different high-level programming languages without translating them into EVM bytecode or intermediate languages.

**Motivations.** A direct executable formal semantics of the high-level smart contract programming language is a must for both understanding and verifying smart contracts. Firstly, programmers write and reason about smart contracts at the level of source code without the semantics of which they are required to understand how Solidity programs are compiled into EVM bytecode in order to understand these contracts, which is far from trivial. In addition, there may be semantic gaps between high-level smart contract programming languages and lowlevel bytecode. Therefore, both high-level [27,48,49,15,11] and low-level [24,21] semantics definitions are necessary to conduct equivalence checking to guarantee that security properties are preserved at both levels and reason about compiler bugs. Secondly, even though smart contracts can be transformed into programs in intermediate languages to be analyzed and verified in existing model checkers and verifiers, the equivalence checking of the high-level smart contract programming language and the intermediate language considered is crucial to the validity of the verification. For instance, most of the false positives reported in Zeus [28] are caused by the semantic inconsistency of the abstract language and Solidity.

As domain-specific languages, high-level smart contract programming languages, such as Solidity, Vyper, Bamboo, etc, intend to implement the correct or desired semantics of smart contracts although they may not actually achieve this. This means that these languages are semantically similar in order to interpret the same high-level semantics of smart contracts. For instance, Vyper is quite similar to Solidity in spite of syntax differences and the semantics interpreted by Bamboo is consistent with that of Solidity (cf. Section 2.1 for details). Considering this fact, we propose a generalized formal semantic framework based on a general semantic model of smart contracts. Different from previous works which either analyze and verify smart contracts on EVM semantics or interpret Solidity semantics with the semantics of intermediate languages, the proposed framework aims to generate a direct executable formal semantics of a particular high-level smart contract programming language to facilitate the high-level verification of contracts and reason about compiler bugs. Furthermore, this framework provides a uniform formal specification of smart contracts, making it possible to apply verification techniques to contracts written in different languages.

**Challenges.** The challenges of developing a generalized formal semantic framework mainly lie in the construction of a general semantic model of smart contracts. Firstly, different high-level smart contract programming languages differ in syntax which limits state transitions. Compared with Solidity, Vyper [8] and Bamboo [1] have more syntax limits to exclude some vulnerabilities reported in Solidity. For instance, Vyper eliminates `gasless send` by blocking recursive calls and infinite loops, and `reentrancy attacks` by excluding the possibility of state changes after external calls [40]. In addition, there are no state variables in Bamboo and each contract represents a particular execution state, making it possible to limit operations to certain states to prevent attacks. Therefore, we need to take into account the syntax differences when constructing a general semantic model for smart contracts. Secondly, semantics developed with the general semantic model must be direct to guarantee the validity of the verification. For instance, as discussed above, even though intermediate languages may be a good solution to construct a general semantic model, they introduce semantic-level equivalence checking issues due to pure syntax translations.

**Contributions.** In this work, we develop a generalized formal semantic framework for smart contracts. The contributions of this work lie in three aspects. Firstly, our work is the first approach, to our knowledge, to a generalized formal semantic framework for smart contracts which can directly handle contracts written in different high-level programming languages. Secondly, a general semantic model of smart contracts is constructed with rewriting logic in the K-framework. With the general semantic model, a direct executable formal semantics of a particular high-level smart contract programming language can be constructed as long as its core features fall into the ones defined in this model. The general semantic model is validated with its interpretation in Solidity using the Solidity compiler test set [6] and evaluation results show that it is complete and correct. Lastly, the generated semantics facilitates the formal verification of smart contracts written in a particular high-level programming language as a formal specification of the corresponding language. Together with low-level specifications [24,21], it allows us to conduct equivalence checking on high-level programs and low-level bytecode to reason about compiler bugs and guarantee that security properties are preserved at both levels.

**Outline.** The remaining part of this paper is organized as follows. In Section 2, we introduce smart contracts and the K-framework. The general semantic model of smart contracts is introduced in Section 3. In Section 4, we take Solidity as an example to illustrate how to generate a direct executable formal semantics of a particular high-level smart contract programming language based on the general semantic model. Section 5 shows the evaluation results of the proposed framework. Section 6 concludes this work.

## 2 Preliminaries

In this section, we briefly introduce smart contracts and the K-framework.

### 2.1 Smart Contracts

**Solidity Smart Contracts.** Ethereum [2,47], proposed in late 2013 by Vitalik Buterin, is a blockchain-based distributed computing platform supporting the functionality of smart contracts. It provides a decentralized international network where each participant node equipped with EVM can execute smart contracts. It also provides a cryptocurrency called "ether" (ETH) which can be transferred between different accounts and used to compensate participant nodes for their computations on smart contracts.

Solidity is one of the high-level programming languages to implement smart contracts on Ethereum. A smart contract written in Solidity can be compiled into EVM bytecode and executed by any participant node equipped with EVM. A Solidity smart contract is a collection of code (its functions) and data (its state) that resides at a specific address on the Ethereum blockchain [7]. Fig. 1 shows an example of Solidity smart contracts, named `Coin`, implementing a very simple cryptocurrency. In line 2, the public state variable `minter` of type `address` is declared to store the address of the minter of the cryptocurrency, i.e., the owner of the smart contract. The constructor, denoted by `constructor()`, is defined in lines 5–7. Once the smart contract is created and deployed[3], its

[3] How to create and deploy a smart contract is out of scope and can be found in: https://solidity.readthedocs.io

```
1  contract Coin {
2     address public minter;
3     mapping (address => uint) public balances;
4
5     constructor() public {
6        minter = msg.sender;
7     }
8
9     function mint(address receiver, uint amount) public {
10       if (msg.sender != minter) return;
11       balances[receiver] += amount;
12    }
13
14    function send(address receiver, uint amount) public {
15       if (balances[msg.sender] < amount) return;
16       balances[msg.sender] -= amount;
17       balances[receiver] += amount;
18    }
19 }
```

**Fig. 1.** Solidity Smart Contract Example constructor is invoked automatically, and `minter` is set to be the address of its creator (owner), represented by the built-in keyword `msg.sender`. In line 3, the public state variable `balances` is declared to store the balances of users. It is of type `mapping`, which can be considered as a hash-table mapping from keys to values. In this example, `balances` maps from a user (represented as an address) to his/her balance (represented as an unsigned integer value). The `mint` function, defined in lines 9–12, is supposed to be invoked only by its owner to mint coins, the number of which is specified by `amount`, for the user located at the `receiver` address. If `mint` is called by anyone except the owner of the contract, nothing will happen because of the guarding `if` statement in line 10. The `send` function, defined in lines 14–18, can be invoked by any user to transfer coins, the number of which is specified by `amount`, to another user located at the `receiver` address. If the balance is not sufficient, nothing will happen because of the guarding `if` statement in line 15; otherwise, the balances of both sides will be updated accordingly.

A blockchain is actually a globally-shared transactional database or ledger. If one wants to make any state change on the blockchain, he or she has to create a so-called *transaction* which has to be accepted and validated by all other participant nodes. Furthermore, once a transaction is applied to the blockchain, no other transactions can alter it. For example, deploying the `Coin` smart contract generates a transaction because the state of the blockchain is going to be changed, i.e., one more smart contract instance will be included. Similarly, any invocation of the function `mint` or `send` also generates a transaction because the state of the contract instance, which is a part of the whole blockchain, is going to be changed. Transactions have to be selected and added into blocks to be appended to the blockchain. This procedure is the so-called *mining*, and the participant nodes are called *miners*.

**Vyper Smart Contracts.** Vyper is a high-level programming language for smart contracts running on EVM. As an alternative to Solidity, Vyper is con-

```
minter: public(address)
balances: map(address, wei_value)

@public
def __init__():
    self.minter = msg.sender

@public
def mint(receiver: address, amount: wei_value):
    if (msg.sender != self.minter): return
    self.balances[receiver] += amount

@public
def send(receiver: address, amount: wei_value):
    if (self.balances[msg.sender] < amount): return
    self.balances[msg.sender] -= amount
    self.balances[receiver] += amount
```

**Fig. 2.** Vyper Smart Contract Example sidered to be more secure by blocking recursive calls and infinite loops to avoid `gasless send`, and excluding the possibility of state changes after external calls to prevent `reentrancy attacks` [40]. Thus, it is more difficult to write vulnerable code in Vyper. In addition, it supports bounds and overflow checking, and strong typing. Particularly, timing features such as block timestamps are supported as types, making it possible to detect the vulnerability of `timestamp dependence` [32] on Vyper semantics. This is not possible on Solidity semantics since Solidity does not support timing features. Apart from security, simplicity is another goal of Vyper. It aims to provide a more human-readable language, and a simpler compiler implementation. An example Vyper smart contract corresponding to the Solidity smart contract illustrated in Fig. 1 is shown in Fig. 2.

**Bamboo Smart Contracts.** Bamboo is another high-level programming language for Ethereum smart contracts. In Bamboo, state variables are eliminated and each contract represents a particular execution state, making state transitions explicit to avoid `reentrancy attacks` by default. This is because operations in functions are limited to certain states. An example Bamboo smart contract which is equivalent to the Solidity smart contract illustrated in Fig. 1 is shown in Fig. 3. In this example, explicit state transitions are applied to strictly limit operations in the constructor to a certain state. To be specific, the default part in the contract `PreCoin` which is equivalent to the constructor in Fig. 1 can only be invoked once, after which the state is always `Coin`. This is consistent with the fact that the constructor of a Solidity smart contract is only invoked once when a new contract instance is created.

**Comparison.** As introduced above, Vyper smart contracts are similar to Solidity smart contracts regardless of the differences in syntax formats. Compared with Solidity, Vyper simply excludes the vulnerabilities reported in Solidity at syntax level. Apart from the syntax differences, explicit state transitions are applied in Bamboo to prevent potential attacks. Despite the limits in syntax and state transitions, high-level smart contract programming languages have a lot in common in semantics due to the fact that they have to be functionally the same.

```
contract PreCoin(address => uint balances){
    default{
       return then become Coin(sender(msg), balances);
    }
}

contract Coin(address minter, address => uint balances){
    case(void mint(address receiver, uint amount)){
       if (sender(msg) != minter)
          return then become Coin(minter, balances);
       balances[receiver] = balances[receiver] + amount;
       return then become Coin(minter, balances);
    }
    case(void send(address receiver, uint amount)){
       if (balances[sender(msg)] < amount)
          return then become Coin(minter, balances);
       balances[sender(msg)] = balances[sender(msg)] - amount;
       balances[receiver] = balances[receiver] + amount;
       return then become Coin(minter, balances);
    }
}
```

**Fig. 3.** Bamboo Smart Contract Example

### 2.2 The K-framework

The K-framework ($\mathbb{K}$) [39] is a rewriting logic [33] based formal *executable* semantics definition framework. The semantics definitions of various programming languages have been developed using $\mathbb{K}$, such as Java [13], C [18], etc. Particularly, an executable semantics of EVM [24], the bytecode language of smart contracts, has been constructed in the K-framework. $\mathbb{K}$ backends, like the Isabelle theory generator, the model checker, and the deductive verifier, can be utilized to prove properties on the semantics and construct verification tools [42].

A language semantics definition in the K-framework consists of three main parts, namely the language syntax, the configuration specified by the developer and a set of rules constructed based on the syntax and the configuration. Given a semantics definition and some source programs, the K-framework executes the source programs based on the semantics definition. In addition, specified properties can be verified by the formal analysis tools in $\mathbb{K}$ backends. We take IMP [37], a simple imperative language, as an example to show how to define a language semantics in the K-framework.

The configuration of the IMP language is shown in Fig. 4. There are only two cells, namely `k` and `state`, in the whole configuration cell `T`. The cells in the configuration are used to store some information related to the program execution. For instance, the cell `k` stores the program for execution `Pgm`, and in the cell `state` a map is used to store the variable state.

$$\left\langle \left\langle \texttt{\$PGM:Pgm} \right\rangle_k \ \left\langle \texttt{.Map} \right\rangle_{state} \right\rangle_T$$

**Fig. 4.** IMP Configuration

Here, we introduce some basic rules in the K-IMP semantics. These rules are `allocate`, `read` and `write`. The syntax of IMP is also given in Fig. 5.

```
Pgm ::= "int" Ids ";" Stmt  Ids ::= List{Id, ","}
AExp ::= Int | Id | "-" Int | AExp "/" AExp  > AExp "+" AExp | "(" AExp ")"
BExp ::= Bool | AExp "<=" AExp | "!" BExp  > BExp "&&" BExp | "(" BExp ")"
Block ::= "{" "}" | "{" Stmt "}"
Stmt ::= Block | Id "=" AExp ";" | "if" "(" BExp ")" Block "else" Block |
"while" "(" BExp ")" Block > Stmt Stmt
```

**Fig. 5.** Syntax of IMP

RULE ALLOCATE

$$\left\langle \frac{\texttt{int X,Xs;S}}{\texttt{int Xs;S}} \cdots \right\rangle_{k} \left\langle \frac{\texttt{Rho:Map}}{\texttt{Rho (X |-> 0)}} \right\rangle_{state}$$

`requires notBool (X in keys(Rho))`

RULE FINISH-ALLOCATE

$$\left\langle \frac{\texttt{int .Ids;S}}{\texttt{S}} \cdots \right\rangle_{k}$$

RULE READ

$$\left\langle \frac{\texttt{X:Id}}{\texttt{I}} \cdots \right\rangle_{k} \left\langle \cdots \ \texttt{X |-> I} \cdots \right\rangle_{state}$$

RULE WRITE

$$\left\langle \frac{\texttt{X = I:Int;}}{\texttt{.}} \cdots \right\rangle_{k} \left\langle \cdots \frac{\texttt{X |-> \_}}{\texttt{X |-> I}} \cdots \right\rangle_{state}$$

Let us start with the rule of memory allocations in IMP shown in ALLOCATE. When `Pgm`, interpreted as `int X,Xs;S`, is encountered, we need to store a list of variables (`X,Xs`) starting from `X` in the cell `state` with a list of mappings. Here `state` can be regarded as a physical memory or storage, and `Xs` is also a list of variables which can be empty. X is popped out of the cell `k` and a new mapping from X to 0 is created in the cell `state`, which means that a memory slot has been allocated for X to store its initial value 0. No duplicate names are allowed in `state`, which is guaranteed by the require condition. Then we go like this until `Xs` becomes empty, which means that all the variables have already been stored in `state`. At this point, the execution of the first part of `Pgm` has been finished and we proceed to the execution of the statement `S`. This can be summarized in FINISH-ALLOCATE where `.Ids` is an empty list of identifiers, which means that the variable list is empty. Please note that `.` means an empty set in the K-framework. If a rule ends with `.`, it means that nothing will be executed.

Then we come to the rules of `read` and `write` for variables. As shown in READ, if we want to look up the value of the variable `X`, we need to search it in the cell `state` by mapping the variable name `X` to its value `I`. So the evaluation of this expression `X` is its value `I`. If we cannot find a mapping for `X`, the program execution will stop at this point. Particularly, `...` means there can be something in the corresponding position. For instance, the mapping of `X` can be in any position in the cell `state`. However, for rules in the cell `k`, `...` can only be at the end since the program which is stored in `k` is executed sequentially. As illustrated in WRITE, if we want to assign the integer `I` to the variable `X`, similarly we need to search it in `state` by mapping the variable name. We also need to rewrite the value of X, denoted by "_" which is a placeholder, to `I`.

Rewriting logic facilitates the construction of a general semantic model for smart contracts. This is because a rewriting logic style semantics consists of a set of rewriting steps from the language syntax to its evaluations. In spite of syntax differences, different smart contract languages have a lot in common in logical

RULE Allocate-General

$$\left\langle \frac{\texttt{\#allocate(X, I)}}{\cdot} \cdots \right\rangle_k \left\langle \frac{\texttt{Rho:Map}}{\texttt{Rho (X |-> I)}} \right\rangle_{state}$$

`requires notBool (X in keys(Rho))`

RULE Read-General

$$\left\langle \frac{\texttt{\#read(X)}}{\texttt{I}} \cdots \right\rangle_k$$
$$\left\langle \cdots \texttt{ X |-> I } \cdots \right\rangle_{state}$$

RULE Write-General

$$\left\langle \frac{\texttt{\#write(X, I)}}{\cdot} \cdots \right\rangle_k \left\langle \cdots \frac{\texttt{X |-> \_}}{\texttt{X |-> I}} \cdots \right\rangle_{state}$$

RULE Allocate-IMP

$$\left\langle \frac{\texttt{int X,Xs;S}}{\texttt{\#allocate(X, 0)} \curvearrowright \texttt{int Xs;S}} \cdots \right\rangle_k$$

RULE Read-IMP

$$\left\langle \frac{\texttt{X:Id}}{\texttt{\#read(X)}} \cdots \right\rangle_k$$

RULE Write-IMP

$$\left\langle \frac{\texttt{X = I:Int;}}{\texttt{\#write(X, I)}} \cdots \right\rangle_k$$

```
MemoryOperations ::= #read(Id) | #write(Id, Int) | #allocate(Id, Int)
```

**Fig. 6.** Syntax of General Memory Operations aspects to achieve the equivalent functionality. Rewriting logic makes it possible to separate the language syntax from the common logical aspects based on which the general semantic model is constructed. The semantics rules introduced above can be general and not specific to IMP. We show the general rules for `read`, `write` and `allocate` in Read-General, Write-General and Allocate-General, respectively. In these rules, `#read`, `#write` and `#allocate` represent the functions to read, write and allocate memory slots for variables with specified parameters and their syntax is shown in Fig. 6. The semantics rules for memory operations in IMP can be obtained by rewriting the corresponding IMP syntax to the general memory operations defined above, namely `#read`, `#write` and `#allocate`, which form a general semantic model. The semantics rules for `read`, `write` and `allocate` in IMP based on the general semantic model are shown in Read-IMP, Write-IMP and Allocate-IMP, respectively. Particularly, the symbol $\curvearrowright$ means "followed by". The semantics rules interpreted with the internal semantics of the general memory operations defined in Fig. 6 are equivalent to those developed from scratch, namely Read, Write and Allocate. Rather than pure syntax translations to intermediate languages, a general semantic model enables semantic-level mappings to commonly shared high-level features.

## 3 A General Semantic Model

Different high-level smart contract programming languages vary in syntax but have a lot in common semantically to achieve the equivalent functionality. Considering this fact, we construct a general semantic model for smart contracts based on the commonly shared high-level semantic features that are independent of any specific language or platform. The semantics of a high-level smart contract programming language can be summarized into three aspects in terms of its functionality, namely memory operations, new contract instance creations and function calls. Particularly, new contract instance creations and function calls are the two kinds of transactions on the blockchain. In this section, we present an overview of the desired semantics of these three core features.

### 3.1 Syntax

The syntax of the general semantic model is defined in the K-framework and shown in Fig. 7. Due to limit of space, we only present the syntax of rewriting steps related to memory operations, new contract instance creations and function calls with `MemOp`, `NewInstanceCreation` and `InstanceStateUpdate`, respectively. Particularly, `ExpressionList` is a list of `Expressions`. `TypeName` consists of `ElementaryTypeName` which takes one memory slot, `ComplexTypeName` which is composed of a set of `ElementaryTypeNames`, and `ReferenceTypeName` which refers to a pre-defined instance. For Solidity, `ElementaryTypeName` consists of all the elementary types defined in the official documentation [7] except `Byte`. `ComplexTypeName` refers to mappings, arrays and `Byte`. `ReferenceTypeName` involves user-defined types and function types. `Id` stands for identifiers. `Int` and `Bool` represent integers and Boolean values, respectively. `Values`, a subset of `ExpressionList`, is a list of `Value` types which can be integers (`Int`) or Boolean types (`Bool`). `Msg` is the type of transaction information. `VarInfo` stores variable information. `MemberAccess` deals with expressions in member access formats.

```
RewritingSteps ::= MemOp | NewInstanceCreation | InstanceStateUpdate

MemOp ::= read(Expression) | readAddress(Int, Id) | write(Expression, Value)
        | writeAddress(Int, Id, Value) | allocate(Int, VarInfo)
        | allocateAddress(Int, Int, Id, Value)

NewInstanceCreation ::= createNewInstance(Id, ExpressionList)
        | updateState(Id) | allocateStorage(Id)
        | initInstance(Id, ExpressionList)

InstanceStateUpdate ::= functionCall(Expression; Expression; Id;
        ExpressionList; Msg) | functionCall(Id; ExpressionList)
        | switchContext(Int, Int, Id, Msg) | returnContext(Int)
        | exception() | updateExceptionState() | revertState()

Expression ::= Id | Value | Msg | VarInfo | MemberAccess
ExpressionList ::= List{Expression, ","} | Values
Value ::= Int | Bool  Values ::= List{Value, ","}
Msg ::= #msgInfo(Int, Int, Int, Int)
VarInfo ::= #varInfo(Id, TypeName, Id, Value)
MemberAccess ::= #memberAccess(Expression, Id)
TypeName ::= ElementaryTypeName | ComplexTypeName | ReferenceTypeName
```

**Fig. 7.** Syntax of the General Semantic Model

### 3.2 Configuration

The runtime configuration indicates program states at each execution step, making detailed runtime features available. The runtime configuration of the general semantic model is illustrated in Fig. 8. Due to limit of space, only a part of the cells is presented here. In this configuration, there are six main cells in the whole configuration cell `T` and they are `k`, `controlStacks`, `contracts`, `functions`, `contractInstances` and `transactions`. The value of each cell is initialized in the configuration with its type specified. A dot followed by any type represents an empty set of this type. For instance, `.List` is an empty list. Particularly, `K` is the most general type which can be any specific type defined in the K-framework.

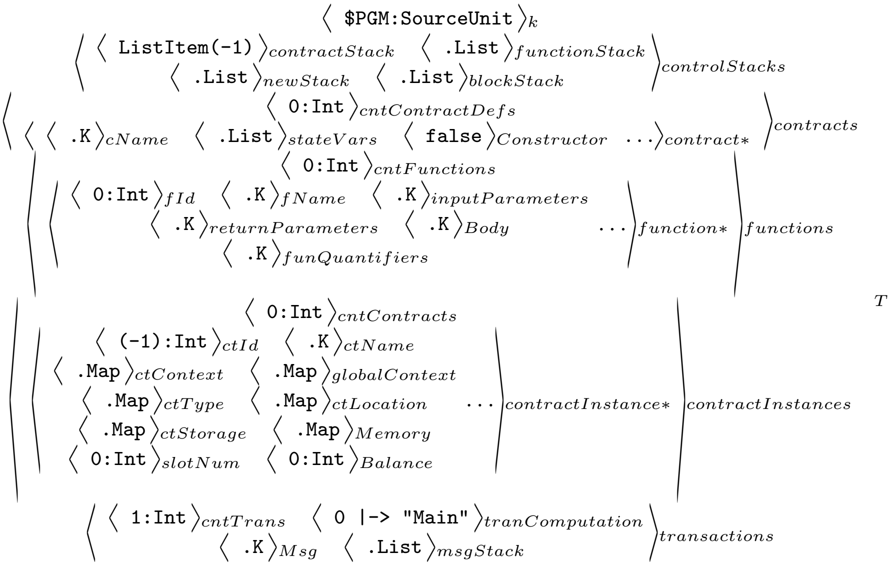

**Fig. 8.** Runtime Configuration of the General Semantic Model

In `k`, source programs, called SourceUnit, are stored for execution. If the programs stored in `k` terminate in a proper way, there will be a dot in this cell, indicating that this cell is empty and there are no more programs to execute.

`controlStacks` consists of `contractStack`, `functionStack`, `newStack` and `blockStack`. To be specific, `contractStack` keeps track of the current contract instance. `functionStack` stores a list of function calls. `newStack` records a list of new contract instance creations. `blockStack` stores a list of variable contexts to look up and assign values to variables in different scopes.

In `contracts`, a set of contract definitions is stored. Each cell `contract` represents a contract definition. The number of distinct contracts is counted in `cntContractDefs`. In `contract`, the contract name is stored in `cName`. State variable information is stored in `stateVars`. In addition, `Constructor` indicates whether the contract has a constructor or not and its initial value is false.

Similarly, `functions` stores a set of function definitions. Each cell `function` represents a function definition. The total number of function definitions is stored in `cntFunctions`. For each function definition, the function Id and the function name are stored in `fId` and `fName`, respectively. In addtion, function parameters, including input parameters and return parameters, are recorded in the corresponding cells. We also store the function body in the cell `Body` and the function quantifiers which can be modifiers or specifiers in the cell `funQuantifiers`.

In `contractInstances`, there is a set of contract instances. Each cell `contractInstance` represents a contract instance. The number of contract instances is counted in `cntContracts`. We store the contract instance Id and the name of its associated contract in the cells `ctId` and `ctName`, respectively. Four different mappings are applied to keep track of more information of a variable. Specifically speaking, `ctContext`, `ctType`, `ctLocation` and `ctStorage/Memory` record the mappings from a variable name to its logical address in the storage or memory, a variable name to its type, a variable name to its location information, namely "global" or "local", and the logical address of a variable in the storage or memory to its value, respectively. `globalContext` keeps track of the state variable context. The number of memory slots taken by variables is calculated in `slotNum`. The cell `Balance` records the balance of each contract instance.

In the cell `transactions`, we keep track of the number of transactions in `cntTrans`, every transaction in `tranComputation` and also "msg" information in `Msg` and `msgStack`. "msg" is a keyword in smart contracts to represent transaction information. For instance, "msg.sender" is the caller of the function and "msg.value" specifies the amount of ether to be transferred in Solidity. The cell `msgStack` stores a list of transaction information tuples while `Msg` records the current one. We simulate transactions of smart contracts with a "Main" contract which is similar to the main function in C. In the "Main" contract, new contract instances can be created and external function calls to these instances are available. The Id of the "Main" contract is "-1", since other contract instances start from 0. Therefore, the initialized content in `contractStack` is `ListItem(-1)`, and `cntTrans` is counted from 1, which means that the creation of the "Main" contract is the first transaction recorded in `tranComputation`.

### 3.3 Semantics of the Core Features

We introduce the semantics rules for the core features in smart contracts. Due to limit of space, the implementation details (cf. [3]) of the sub-steps are omitted.

**Memory Operations.** We present an overview of the semantics rules for memory operations on elementary types, such as `int`, `uint` and `address` in Solidity, each of which takes only one memory slot. Complex types, such as arrays, mappings, etc, are compositions of elementary types. A memory operation on a complex type can be regarded as a set of recursive memory operations on elementary types. For instance, the memory allocation for a one-dimensional fixed-size array is equivalent to allocating an elementary type for each index of this array. Reading and writing a particular index involve recursive steps to retrieve the logical address of this index from the base address of the array. Mappings are similar to dynamic arrays. For a mapping from `address` to `uint`, the memory allocation for this mapping is equivalent to allocating an unsigned integer type at each address involved. Reference types which refer to pre-defined instances can be simply implemented as mappings in the K-framework.

RULE READ

$$\left\langle \frac{\texttt{read(X:Id)}}{\texttt{readAddress(Addr, L)}} \cdots \right\rangle_{k} \quad \left\langle \texttt{ListItem(N:Int)} \cdots \right\rangle_{contractStack}$$

$$\left\langle \begin{array}{l} \left\langle \texttt{N} \right\rangle_{ctId} \quad \left\langle \cdots \texttt{X |-> Addr} \cdots \right\rangle_{ctContext} \\ \quad \left\langle \cdots \texttt{X |-> L} \cdots \right\rangle_{ctLocation} \\ \left\langle \cdots \texttt{X |-> T:ElementaryTypeName} \cdots \right\rangle_{ctType} \end{array} \cdots \right\rangle_{contractInstance}$$

RULE WRITE

$$\left\langle \frac{\texttt{write(X:Id, V:Value)}}{\texttt{writeAddress(Addr, L, V)}} \cdots \right\rangle_{k} \quad \left\langle \texttt{ListItem(N:Int)} \cdots \right\rangle_{contractStack}$$

$$\left\langle \begin{array}{l} \left\langle \texttt{N} \right\rangle_{ctId} \quad \left\langle \cdots \texttt{X |-> Addr} \cdots \right\rangle_{ctContext} \\ \quad \left\langle \cdots \texttt{X |-> L} \cdots \right\rangle_{ctLocation} \\ \left\langle \cdots \texttt{X |-> T:ElementaryTypeName} \cdots \right\rangle_{ctType} \end{array} \cdots \right\rangle_{contractInstance}$$

RULE ALLOCATE

$$\left\langle \frac{\texttt{allocate(N:Int, \#varInfo(X:Id, T:ElementaryTypeName, L:Id, V:Value))}}{\texttt{allocateAddress(N, Addr, L, V)}} \cdots \right\rangle_{k}$$

$$\left\langle \begin{array}{l} \left\langle \texttt{N} \right\rangle_{ctId} \quad \left\langle \frac{\texttt{Addr}}{\texttt{Addr +Int 1}} \right\rangle_{slotNum} \quad \left\langle \frac{\texttt{TYPE:Map}}{\texttt{TYPE (X |-> T)}} \right\rangle_{ctType} \\ \left\langle \frac{\texttt{CON:Map}}{\texttt{CON (X |-> Addr)}} \right\rangle_{ctContext} \quad \left\langle \frac{\texttt{LOC:Map}}{\texttt{LOC (X |-> L)}} \right\rangle_{ctLocation} \end{array} \cdots \right\rangle_{contractInstance}$$

RULE NEW-CONTRACT-INSTANCE-CREATION

$$\left\langle \frac{\texttt{createNewInstance(X:Id, E:ExpressionList)}}{\texttt{updateState(X)} \curvearrowright \texttt{allocateStorage(X)} \curvearrowright \texttt{initInstance(X, E)}} \cdots \right\rangle_{k}$$

RULE FUNCTION-CALL

$$\left\langle \frac{\texttt{functionCall(C:Int; R:Int; F:Id; Es:Values; M:Msg)}}{\texttt{switchContext(C, R, F, M)} \curvearrowright \texttt{functionCall(F; Es)} \curvearrowright \texttt{returnContext(R)}} \cdots \right\rangle_{k}$$

Let us start with the `read` operation on elementary types shown in READ. Here, we consider the object `X` as a variable which is an `Id` type. The first thing to do is to get the current execution context. This is achieved by retrieving the current contract instance Id `N` in `contractStack` and mapping the corresponding contract instance with `N` in the cell `ctId`. After that, we retrieve the logical address of `X`, denoted by `Addr`, in `ctContext` and the location information of `X`, denoted by `L`, in `ctLocation`. With these two parameters, we can obtain the evaluation of `X` through `readAddress` which retrieves the value located at `Addr` in the associated cell specified by `L`. To be specific, if `L` specifies this variable as a global one, the search space is `ctStorage`. Otherwise, the value is retrieved in `Memory`. `write` is similar to `read`. After retrieving the logical address of `X`, denoted by `Addr`, and the location information of `X`, denoted by `L`, we rewrite the value at `Addr` to the value `V` in the cell specified by `L` through `writeAddress`.

Then we come to the allocation for elementary types shown in ALLOCATE. The first input parameter `N` indicates the object contract instance Id. The variable information including the name `X`, the type `T`, the location information `L` and the initial value `V`, is stored in `#varInfo`. First, we retrieve the corresponding instance by mapping the Id `N` in `ctId`. Then the number of memory slots is increased by 1 in `slotNum`. After that, the variable information is recorded in the associated cells. To be specific, we record the logical address `Addr`, the type `T`, and the location information `L` in `ctContext`, `ctType` and `ctLocation`, respectively. Finally, a memory slot is allocated for this variable through `allocateAddress`.

**New Contract Instance Creations.** As illustrated in New-Contract-Instance-Creation, the contract name `X` and the arguments in the constructor `E` are taken as input parameters to create a new instance of `X`. There are altogether three sub-steps for this transaction and they are `updateState`, `allocateStorage` and `initInstance`. To be specific, `updateState` updates the blockchain states, including the states of contract instances and transactions, and the stack information to indicate the new contract instance creation. In addition, `allocateStorage` allocates state variables and `initInstance` deals with initialization issues, such as calling the constructor, in the new instance.

**Function Calls.** In order to make the semantics of function calls general for all kinds of calls and extensible for different smart contract languages, a uniform format is applied to generalize the semantics. The uniform format is `functionCall(Id_of_Caller; Id_of_Recipient; Function_Name; Arguments; Msg_Info)`. Particularly, `Msg_Info` represents the transaction information, including the Ids of the caller and the recipient instances, the value of digital assets to be transferred and the transaction fees to be consumed. The semantics rule for function calls based on this format is shown in Function-Call.

In the rule Function-Call, the caller of this function is `C` and the recipient is `R`. `F` is the function name and `Es` specifies the function call arguments. `M` is the "msg" information to keep track of transactions. In particular, the types of these parameters have been specified. The semantics of function calls is designed from a general point of view. Each external function call is regarded as an extension of an internal function call. Whenever there is an external function call, we first switch to the recipient instance and then call the function in this instance as an internal call. Finally, we switch back to the caller instance. In this way, external function calls can be achieved through internal function calls and switches of contract instances. This mechanism also applies to internal function calls where the caller is the same as the recipient. There are three sub-steps in Function-Call. The first one is to switch to the recipient instance from the caller through `switchContext`. The second is an internal function call `functionCall`. The last one is to return to the caller instance through `returnContext`.

Particularly, the semantics of function calls is equipped with exception handling features. If an exception is encountered, it will be propagated to the transactional function call to revert the whole transaction. The propagation of exceptions is a sub-step in `returnContext`. The exception handling mechanism is also general, making it possible to deal with all kinds of exception handling features in smart contracts, such as `revert` and `assert` in Solidity, in a similar way.

RULE Exception-Propagation

$$\left\langle \frac{\texttt{exception()}}{\texttt{updateExceptionState()}} \cdots \right\rangle_{k} \left\langle \texttt{ListItem(R)ListItem(C)} \ldots \right\rangle_{contractStack}$$

`requires C >=Int 0`

RULE Transaction-Reversion

$$\left\langle \begin{array}{l} \dfrac{\texttt{exception()}}{\texttt{updateExceptionState()}} \ldots \\ \quad \curvearrowright \texttt{revertState()} \end{array} \right\rangle_{k} \left\langle \texttt{ListItem(R)ListItem(-1)} \right\rangle_{contractStack}$$

There are two stages in handling exceptions. The first one is the propagation of exceptions to the transactional function call as shown in Exception-Propagation, and the second is the reversion of the transaction as shown in Transaction-Reversion. The first stage is present in nested calls to propagate exceptions to the transactional function call, while the second stage is only present in the transactional function call stemming from the "Main" contract. In the stage of propagating exceptions, the exception state is updated through `updateExceptionState()` to indicate that an exception has been encountered. Particularly, the Id of the caller instance should be larger than or equal to 0 since the caller cannot be the "Main" contract. And in the stage of reverting transactions, the caller is the "Main" contract whose Id is "-1". In addition to updating the exception state, the whole transaction is reverted through `revertState()`.

## 4 Direct Semantics Generation

A direct semantics of a high-level smart contract programming language can be developed based on the general semantic model introduced above. From the perspective of rewriting logic, a language semantics is a set of rewriting steps from the language syntax to its evaluations. Each of these rewriting steps implements a function to move the syntax a step further to its final evaluations. The general semantic model which consists of a set of internal rewriting steps and defines the desired semantics of smart contracts can be regarded as a logical intermediate language. A direct semantics of a high-level smart contract programming language can be constructed by rewriting its syntax to the features in the general semantic model with several functional steps. This also indicates the process of smart contract language design. We take Solidity as an example to illustrate how to generate the semantics based on the general semantic model. The semantics rules presented below are based on the Solidity syntax defined in [7].

Let us start with the `look-up` operation in Solidity. As shown in Look-Up, the object is considered to be a variable `X`. `X` is evaluated with `read` in the general semantic model. We simply rewrite the corresponding Solidity syntax to `read`. `assignment` is similar to `look-up`. As shown in Assignment, we simply rewrite the assignment syntax in Solidity to `write` in the general semantic model.

RULE Look-Up RULE Assignment RULE New-Instance-Solidity

$$\left\langle \frac{\texttt{X:Id}}{\texttt{read(X)}} \cdots \right\rangle_k \quad \left\langle \frac{\texttt{X:Id = V:Value}}{\texttt{write(X, V)}} \cdots \right\rangle_k \quad \left\langle \frac{\texttt{new X:Id (E:ExpressionList)}}{\texttt{createNewInstance(X, E)}} \cdots \right\rangle_k$$

Both state and local variable allocations are achieved through `allocate` in the general semantic model. State variables are allocated when new contract instances are created, while local variables are allocated right after declarations.

In New-Instance-Solidity, the syntax of new contract instance creations in Solidity is rewritten to `createNewInstance` in the general semantic model.

Function calls in Solidity are written in a format similar to member access. For instance, `target.deposit.value(2)()` is a typical function call in Solidity. To be specific, `target` specifies the recipient instance and `deposit` is the function

RULE FUNCTION-CALL-SOLIDITY

$$\left\langle \frac{\texttt{\#memberAccess(R:Int, F:Id)} \curvearrowright \texttt{Es:Values} \curvearrowright \texttt{MsgValue:Int} \curvearrowright \texttt{MsgGas:Int}}{\texttt{functionCall(C; R; F; Es; \#msgInfo(C, R, MsgValue, MsgGas))}} \cdots \right\rangle_k$$

$$\left\langle \texttt{ListItem(C:Int)} \ldots \right\rangle_{contractStack}$$

RULE REVERT

$$\left\langle \frac{\texttt{revert(.ExpressionList);}}{\texttt{exception()}} \cdots \right\rangle_k$$

RULE ASSERT

$$\left\langle \frac{\texttt{assert(true);}}{\cdot} \cdots \right\rangle_k \quad \left\langle \frac{\texttt{assert(false);}}{\texttt{exception()}} \cdots \right\rangle_k$$

RULE REQUIRE

$$\left\langle \frac{\texttt{require(true);}}{\cdot} \cdots \right\rangle_k \quad \left\langle \frac{\texttt{require(false);}}{\texttt{exception()}} \cdots \right\rangle_k$$

to be called in that instance. `value` specifies `msg.value` as 2. In addition, we can specify other parameters, such as `msg.gas`, function arguments, etc. When it comes to the semantics of function calls in Solidity, the first thing to do is to decompose the member access like format and transform it into the one in the general semantic model. As shown in FUNCTION-CALL-SOLIDITY, each decomposed part in Solidity calls is reorganized in `functionCall`. Specifically speaking, `#memberAccess(R:Int, F:Id)` specifies the recipient instance `R` and the function to be called in this instance `F`. `Es` specifies the function arguments. `MsgValue` and `MsgGas` represent `msg.value` and `msg.gas`, respectively.

The semantics rules for function calls apply to all kinds of function calls in Solidity, including high-level and low-level calls, constructors and fallback functions. For instance, if there is no function name specified in a function call or the specified function name does not match any existing function in the recipient instance, the first decomposed part in FUNCTION-CALL-SOLIDITY will be `#memberAccess(R:Int, String2Id("fallback"))` where `R` is the Id of the recipient instance and "fallback" refers to the fallback function in that instance. In this case, the fallback function in `R` will be invoked. In addition, in the case of `delegatecall`, the recipient instance `R` is the same as the caller instance `C` since the execution takes place in the caller's context.

Exception handling features in Solidity can be interpreted with the semantics of `exception()` in the general semantic model. The semantics rules for `revert`, `assert` and `require` are shown in REVERT, ASSERT and REQUIRE, respectively.

## 5 Evaluation

We evaluate the proposed generalized formal semantic framework for smart contracts by showing that the generated semantics, an interpretation of the general semantic model with a particular language, is consistent with the semantics interpreted by the corresponding official compiler on benchmarks. The testing language makes no difference to the evaluation since it aims to validate the semantics of the commonly shared high-level features defined in the general semantic model. We take Solidity as an object for the evaluation since there are sufficient Solidity smart contracts available for testing the generated Solidity

**Table 1.** Coverage of the Generated Solidity Semantics

| Features | Coverage | Features | Coverage |
|---|---|---|---|
| **Types(Core)** | | **Statements(Core)** | |
| *Elementary Types* | | `If Statement` | FC |
| `address` | FC | `While Statement` | FC |
| `bool` | FC | `For Statement` | FC |
| `string` | FC | `Block` | FC |
| `Int` | FC | `Inline Assembly Statement` | N |
| `Uint` | FC | | |
| `Byte` | FC | `Do While Statement` | FC |
| `Fixed` | N | `Place Holder Statement` | FC |
| `Ufixed` | N | `Continue` | FC |
| *User-defined Types* | FC | `Break` | FC |
| *Mappings* | FC | `Return` | FC |
| *Array Types* | FC | `Throw,Revert,Assert,Require` | FC |
| *Function Types* | FC | `Simple Statement` | FC |
| *address payable* | FC | `Emit Statement` | FC |
| **Functions(Core)** | | **Expressions(Core)** | |
| *Function Definitions* | | `Bitwise Operations` | FC |
| `Constructors` | FC | `Arithmetic Operations` | FC |
| `Normal Functions` | FC | `Logical Operations` | FC |
| `Fallback Functions` | FC | `Comparison Operations` | FC |
| `Modifiers` | FC | `Assignment` | FC |
| *Function Calls* | | `Look Up` | FC |
| `Internal Function Calls` | FC | `New Expression` | FC |
| `External Function Calls` | FC | `Other Expressions` | FC |
| **Using For** | FC | **Inheritance** | FC |
| **Event** | FC | | |

FC: Fully Covered and Consistent with Solidity IDE    N: Not Covered semantics. The Solidity semantics developed with the proposed framework is publicly available at https://github.com/kframework/solidity-semantics.

The generated Solidity semantics is evaluated from two perspectives: the first one is its coverage (i.e., completeness), and the second is its correctness (i.e., consistency with Solidity compilers). Evaluation results show that the Solidity semantics developed with the proposed framework completely covers the supported high-level core language features specified by the official Solidity documentation [7] and is consistent with the official Solidity compiler Remix [5].

We evaluate and test the Solidity semantics developed with the proposed framework with the Solidity compiler test set [6]. This test set is regarded as a standard test set or benchmarks for evaluating Solidity semantics since the test programs are written in a standard or correct way defined by the language developers and cover all the features in Solidity. There are altogether 482 tests in the Solidity compiler test set. The evaluation is done by manually comparing the execution behaviours of the generated Solidity semantics with the ones of the Remix compiler on the test programs. We consider the generated Solidity semantics is correct if the execution behaviours indicated in the configuration are consistent with the ones of the Remix compiler. A feature is considered to be fully covered if all the compiler tests involving this feature are passed. We list the coverage of the generated Solidity semantics in Table 1 from the perspective of each feature specified by the official documentation.

From Table 1, we can observe that the generated Solidity semantics completely covers the supported high-level core features of Solidity. As for types, the generated Solidity semantics covers the following elementary types: `address`, `bool`, `string`, `Int`, `Uint` and `Byte`. `Fixed` and `Ufixed` are not covered because they are not fully supported by Solidity yet [7]. User-defined types, including `struct`, contract types and `enum`, are covered. Mappings, arrays, function types and `address payable` are also covered. In addition, the semantics associated with functions, such as function definitions and function calls, is fully covered. The semantics of statements is completely covered except that of `inline assembly statements` which are considered to be low-level features accessing EVM (i.e., this part of semantics can be integrated with KEVM [24]). All kinds of expressions in Solidity are covered. Lastly, the semantics of `event` is also covered and the parts of semantics for `using for` and `inheritance` are covered with rewriting. For all the parts of covered semantics, they are considered to be correct since the execution behaviours involved are consistent with the ones of Remix. Therefore, the generated Solidity semantics can be considered to be complete and correct in terms of the supported high-level core features of Solidity, indicating the completeness and correctness of the general semantic model.

**Threats to Validity.** We validate the general semantic model with its interpretation in Solidity. The validity of the proposed framework holds for any particular high-level smart contract programming language as long as its core features fall into or can be properly rewritten to the ones defined in the general semantic model. The proposed framework may not work if the core features cannot be interpreted with the ones defined in the general semantic model. However, this is unlikely due to the nature of smart contract executions. For instance, transactions in existing instances are implemented with or can be transformed into function calls regardless of the platforms of smart contract programs.

## 6 Conclusion

In this paper, we propose a generalized formal semantic framework for smart contracts. This framework can directly handle smart contracts written in different high-level programming languages, such as Solidity, Vyper, Bamboo, etc, without translating them into EVM bytecode or intermediate languages. In this framework, a direct executable formal semantics of a particular high-level smart contract programming language is constructed based on a general semantic model with rewriting logic. The general semantic model is validated with its interpretation in Solidity and evaluation results show that it is complete and correct. Furthermore, the proposed framework provides a formal specification of smart contracts written in different languages.

**Acknowledgements.** This work is supported by the Ministry of Education, Singapore under its Tier-2 Project (Award Number: MOE2018-T2-1-068) and partially supported by the National Research Foundation, Singapore under its NSoE Programme (Award Number: NSOE-TSS2019-03).
